# Decreased Plasma Histidine Level Predicts Risk of Relapse in Patients with Ulcerative Colitis in Remission

**DOI:** 10.1371/journal.pone.0140716

**Published:** 2015-10-16

**Authors:** Tadakazu Hisamatsu, Nobukazu Ono, Akira Imaizumi, Maiko Mori, Hiroaki Suzuki, Michihide Uo, Masaki Hashimoto, Makoto Naganuma, Katsuyoshi Matsuoka, Shinta Mizuno, Mina T. Kitazume, Tomoharu Yajima, Haruhiko Ogata, Yasushi Iwao, Toshifumi Hibi, Takanori Kanai

**Affiliations:** 1 Department of Internal Medicine, School of Medicine, Keio University, Tokyo, Japan; 2 Institute for Innovation, Ajinomoto Co. Inc., Kawasaki, Japan; 3 Research Institute, Ajinomoto Pharmaceuticals Co. Ltd., Kawasaki, Japan; 4 Center for Diagnostic and Therapeutic Endoscopy, Keio University, Tokyo, Japan; 5 Center for Preventive Medicine, School of Medicine, Keio University, Tokyo, Japan; 6 Center for Advanced IBD Research and Treatment, Kitasato University Kitasato Institute Hospital, Tokyo, Japan; 7 Division of Gastroenterology and Hepatology, The Third Department of Internal Medicine, Kyorin University School of Medicine, Tokyo, Japan; Massachusetts General Hospital, UNITED STATES

## Abstract

Ulcerative colitis (UC) is characterized by chronic intestinal inflammation. Patients with UC have repeated remission and relapse. Clinical biomarkers that can predict relapse in UC patients in remission have not been identified. To facilitate the prediction of relapse of UC, we investigated the potential of novel multivariate indexes using statistical modeling of plasma free amino acid (PFAA) concentrations. We measured fasting PFAA concentrations in 369 UC patients in clinical remission, and 355 were observed prospectively for up to 1 year. Relapse rate within 1 year was 23% (82 of 355 patients). The age- and gender-adjusted hazard ratio for the lowest quartile compared with the highest quartile of plasma histidine concentration was 2.55 (95% confidence interval: 1.41–4.62; p = 0.0020 (log-rank), p for trend = 0.0005). We demonstrated that plasma amino acid profiles in UC patients in clinical remission can predict the risk of relapse within 1 year. Decreased histidine level in PFAAs was associated with increased risk of relapse. Metabolomics could be promising for the establishment of a non-invasive predictive marker in inflammatory bowel disease.

## Introduction

Inflammatory bowel disease (IBD) is a chronic intestinal disorder comprising two major types, Crohn’s disease (CD) and ulcerative colitis (UC) [1]. The colon is mainly involved in UC, while the entire gastrointestinal tract is the target in CD. Patients with UC have repeated remission and relapse, and there are a limited number of clinical biomarkers that can predict relapse in UC patients in remission. Successful development of a predictive marker for relapse would make it possible to modify clinical management, such as de-escalation or escalation of maintenance therapy, and decide when to perform colonoscopy. Recently, endoscopic remission (also known as mucosal healing) has been considered as an ideal treatment goal and predictive factor for long-term prognosis [2, 3], while frequent colonoscopy is a burden for patients and physicians. As a result, development of non-invasive biomarkers that can predict relapse and identify high-risk patients has become desirable.

As a post-genomic technique, metabolomics provide new opportunities to develop novel biomarkers in many diseases [4], including cardiovascular disease [5], diabetes [6], cancer [7], and IBD [8]. Several small studies have used metabolomics to analyze blood or urine samples in patients with IBD. Stephens et al. reported the alteration of urinary nuclear magnetic resonance (NMR) metabolomics profiles between IBD patients and healthy controls [9]. Ooi et al. analyzed gas chromatography/mass spectrometry (GC/MS)-based profiling of serum amino acids and tricarboxylic acid cycle (TCA)-cycle-related molecules in 13 UC and 21 CD patients [10]. Zang et al. used serum ^1^H-NMR-based spectroscopy to investigate metabolic alterations in 20 patients with active UC [11]. Williams et al. also investigated serum metabolic profiling using ^1^H-NMR-based spectroscopy in 44 patients in remission from IBD (24 CD and 20 UC) [12].

Most studies have been performed to compare metabolic alterations between IBD patients and healthy controls, or patients with active disease and those in remission, and have concluded that metabolomics are useful to distinguish IBD from non-IBD. The results obtained from these studies may reflect the pathophysiology of IBD. However, the most valuable roles of metabolomics in IBD clinics are to develop a monitoring marker that reflects disease activity and a prognostic marker regarding the risk of relapse or therapeutic efficacy. The search for a prognostic marker using metabolomics has not pursued sufficiently and it is recognized as an important task in IBD clinics. Previously, we reported (165 patients with CD, 222 with UC, and 210 healthy controls) that plasma aminograms combined with multivariate indices discriminated between CD/UC patients and age-matched healthy controls, as well as between patients with active disease and those in remission. In particular, plasma histidine level was significantly decreased in IBD patients with active disease [13].

The aim of this study was to develop a prognostic factor using metabolomics. We analyzed plasma free amino acid (PFAA) concentrations in UC patients in clinical remission and observed them prospectively for up to 1 year. Decreased histidine in PFAAs in the patients in remission was associated with increased risk of relapse.

## Materials and Methods

### Patients

This study was conducted in accordance with the Declaration of Helsinki, and the protocol was approved by the ethics committees of Keio University School of Medicine. Signed informed consent forms were obtained from all patients and all data were analyzed anonymously throughout the study. The diagnosis of UC was based on established clinical, radiographic, endoscopic and histopathological criteria. Patients’ characteristics were determined from medical records, questionnaires, and interviews. Disease activity was assessed by Lichtiger Clinical Activity Index (CAI). Active was defined as CAI ≥5 for patients with UC. Remission was defined as CAI <5 for patients with UC. Patient inclusion criteria were as follows: (1) diagnosis of UC based on established clinical, radiographic, endoscopic and histopathological criteria; and (2) clinical remission was defined as CAI <5. The baseline characteristics of the patients are summarized in Table 1. A total of 369 UC patients who met the inclusion criteria were included.

**Table 1:** Baseline characteristics of eligible UC patients.

	Non-relapse	Relapse
**n**	273	82
**Gender (M/F)**	124/149	34/48
**Age (yr)**	45±14	45±14
**Duration (years before entry)**	12±9	10±8
**CAI (0/1/2/3/4)**	109/91/36/26/9	20/26/15/14/7
**Remission maintenance therapy**		
**5-ASA**	168	57
**SASP**	99	24
**Steroids**	10	12
**Immunosuppresants**	75	26
**FK506**	4	3

5-ASA, 5-aminosalicylic acid; F, female; M, male; SASP, sulfasalazine.

### Follow-up

Outpatients in remission were reviewed every 2–3 months up to 1 year after enrollment. Clinical activity was evaluated by CAI. Relapse was defined as CAI ≥5 or necessity for additional treatment. Three hundred and fifty-five of 369 patients were observed prospectively for up to 1 year.

### Plasma amino acid analysis

All plasma samples for amino acid analysis were obtained with use of EDTA as the anticoagulant. Plasma was prepared by centrifugation at 3,000 rpm at 4°C for 15 min and then stored at −80°C until analysis. The plasma samples were deproteinized using acetonitrile at a final concentration of 80% before measurement. The amino acid concentrations in the plasma were measured by high-performance liquid chromatography electrospray ionization mass spectrometry, followed by pre-column derivatization. The analytical methods used were as described previously [14–16]. The following 19 amino acids and related molecules were measured and analyzed: alanine, arginine, asparagine, citruline, glutamine, glycine, histidine, isoleucine, leucine, lysine, methionine, ornithine, phenylalanine, proline, serine, threonine, tryptophan, tyrosine and valine.

### Cytokine assay

For the measurement of TNF-alpha, human TNF-alpha Quantkine HS ELISA (R&D systems, Minneapolis, MN) was used according to the manufacturer’s instructions.

### Statistical analysis

Three types of PFAA profiles were used in this study: (1) absolute concentration of each amino acid; (2) gender- and age-adjusted concentration of each amino acid; and (3) normalized data by Box—Cox transformation after gender- and age-adjustment of each amino acid. Cox proportional regression analysis was conducted to calculate hazard ratios (HR) with levels of significance of each amino acid concentration for relapse of UC. The difference in relapse rate of each quartile of amino acid concentration was evaluated by log rank test for trend. The level of significance was set at p<0.05. MATLAB (Mathworks, Natick, MA, USA) and GraphPad Prism (GraphPad Software, La Jolla, CA, USA) were used for statistical analysis.

## Results

### Relapse rate up to 1 year

**Fig 1:**
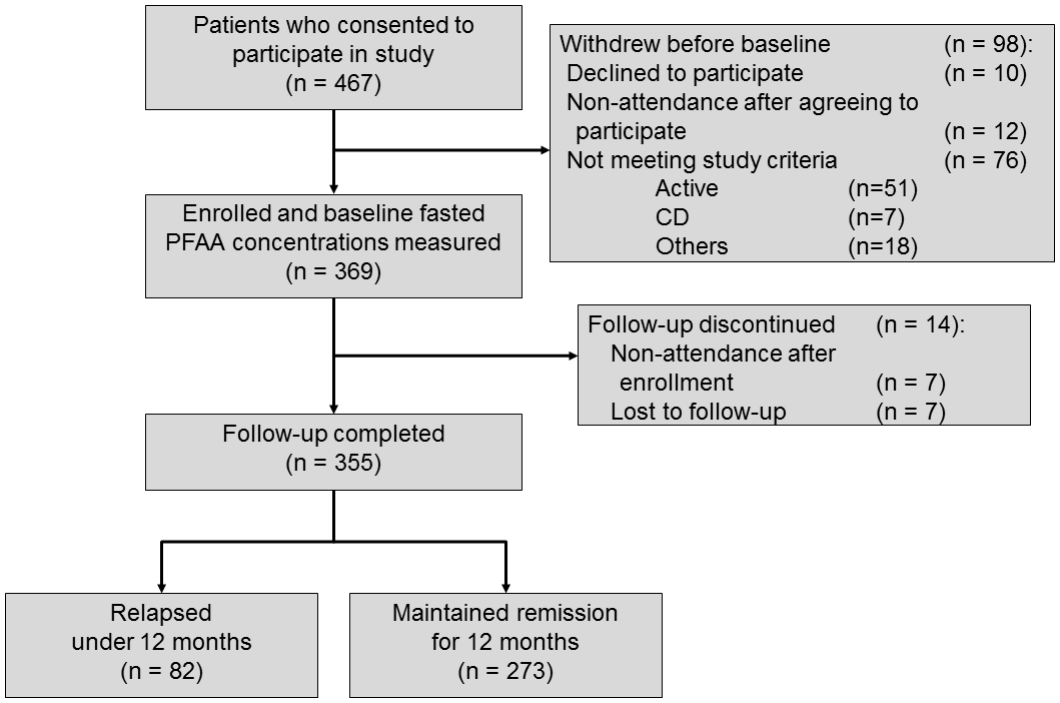
Flowchart of the patients in the study.

**Fig 2:**
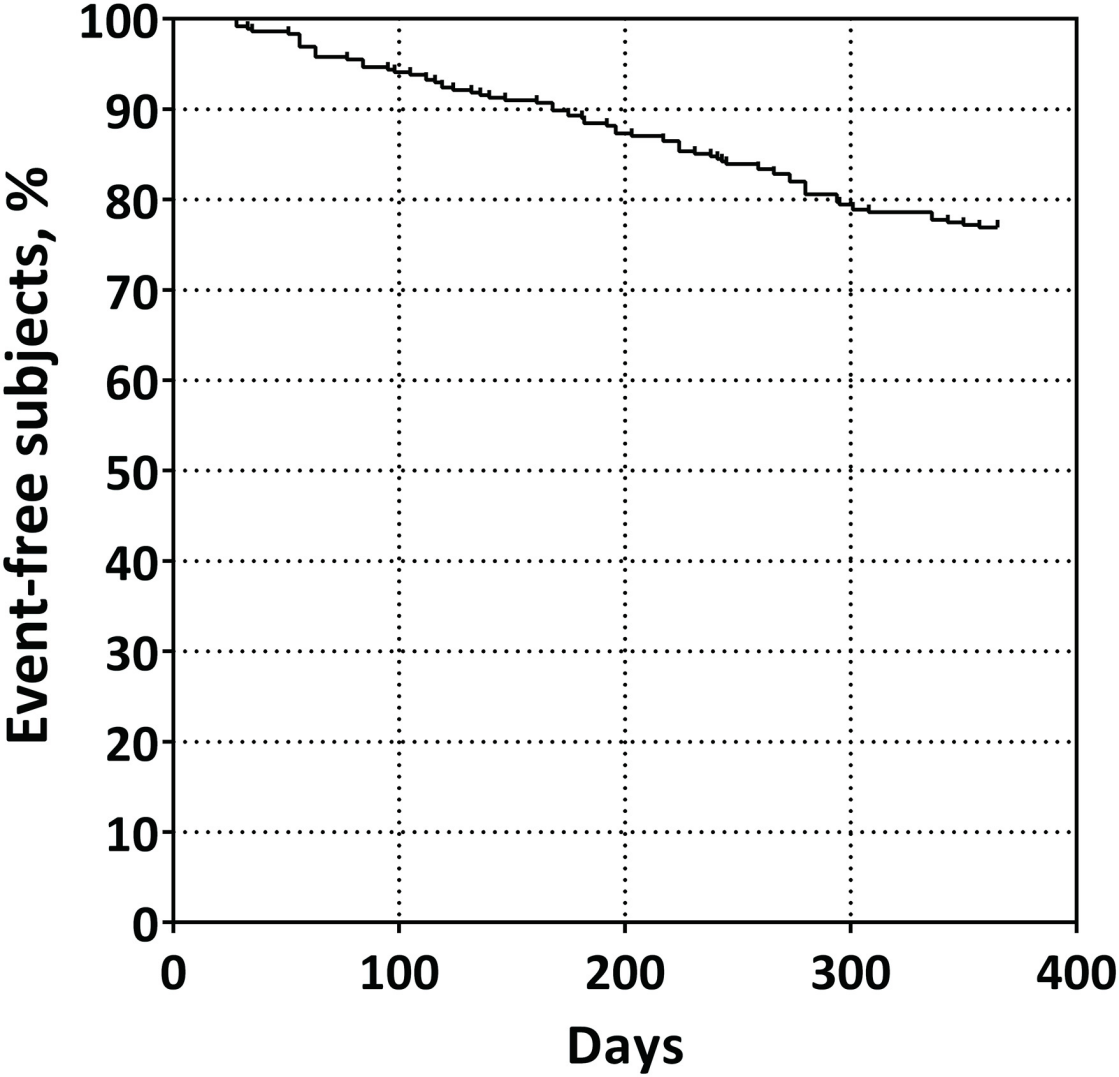
Kaplan—Meier analysis of whole patients who have successfully followed up.

We obtained informed consent from 467 UC patients in remission. Ninety-eight patients were withdrawn before baseline (10 declined to participate, 12 failed to attended after agreeing to participate, and 76 did not meet the study criteria). A total of 369 UC patients in remission were enrolled and we measured fasted PFAA concentrations. During 1 year follow-up, 14 patients were withdrawn. Finally, 355 patients were successfully followed up and analyzed. Within 1 year after enrollment, 82 patients had relapsed (relapse rate 23%, Fig 1). Kaplan—Meier analysis is shown in Fig 2.

### Plasma level of histidine as predictor for relapse of UC

**Fig 3:**
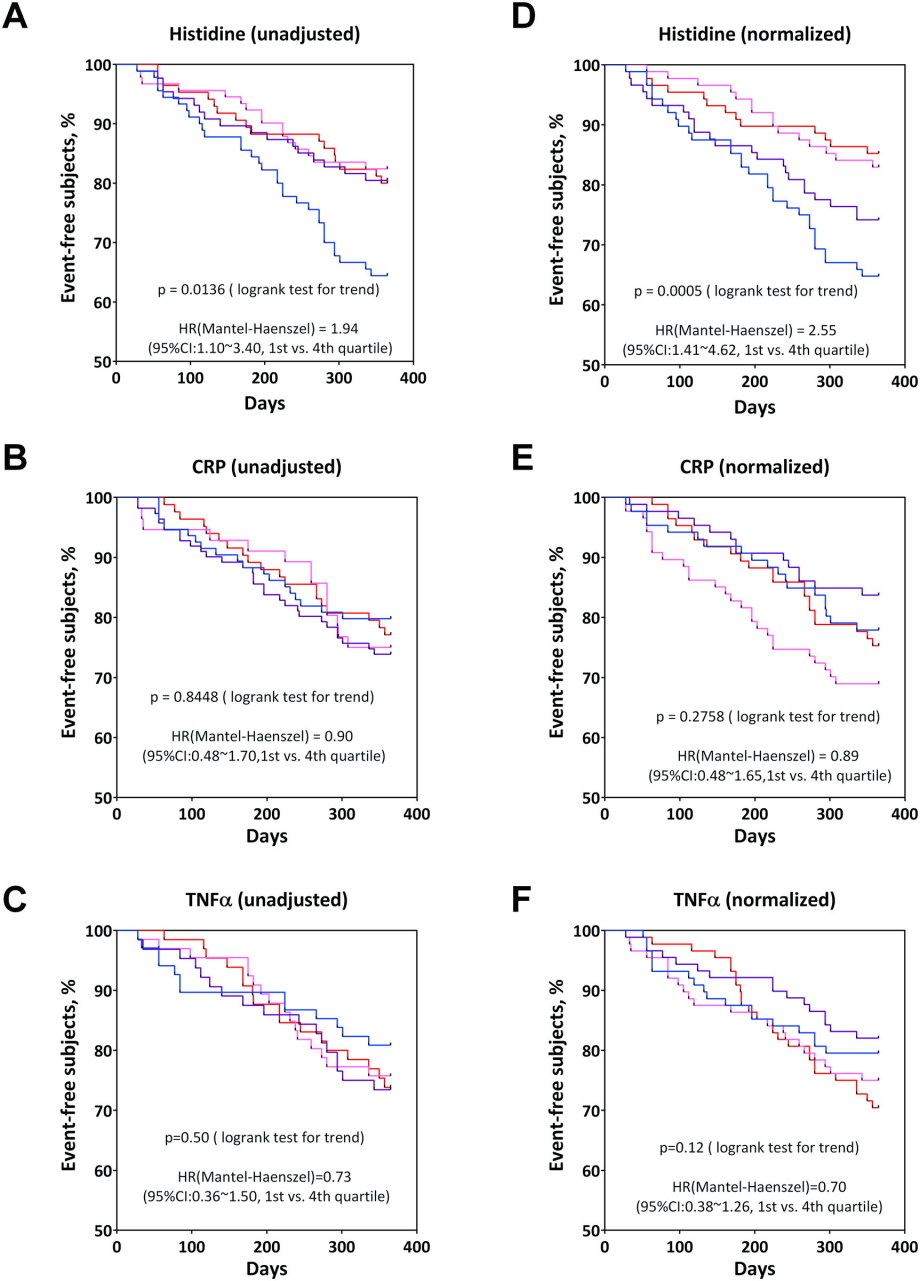
Trends of cumulative relapse ratio for each quartile by unadjusted plasma histidine level (A), unadjusted CRP level (B), unadjusted TNF-alpha level (C), normalized histidine level (D), normalized CRP level (E), and normalized TNF-alpha level (F). Blue: 1^st^ quartile, purple: 2^nd^ quartile, pink: 3^rd^ quartile, red: 4^th^ quartile.

For Cox regression analysis, three types of PFAA profiles were used as described in *Materials and Methods*. Under any conditions of Cox regression analysis, significant HRs were observed only for histidine (p<0.05) even after adjustment and normalization for relapse of UC, while with unadjusted raw data, and gender- and age-adjusted data, significant HRs were observed also for glutamate (p<0.05) (Table 2). The concentration of histidine was inversely correlated with relapse rate of UC (β = −0.2687) (Table 2). In contrast, there were no significant HRs under any conditions for C-reactive protein (CRP; p>0.491) and TNF-alpha (p>0.0625) (Table 2). To confirm the negative dependency of plasma histidine concentration for relapse, Kaplan—Meier curve of each quartile was plotted using raw data, and gender- and age-adjusted data. In any case, a significant negative trend in relapse rate for UC was observed in plasma histidine level (p = 0.0136) (Fig 3A), whereas no significant trend was observed in CRP (p = 0.8448, unadjusted; p = 0.2758, adjusted) and TNF-alpha levels (p = 0.50, unadjusted; p = 0.12, adjusted) (Fig 3B, 3C, 3E and 3F). Note that dependency on concentration was observed in gender- and age-adjusted data more clearly (p = 0.0005) (Fig 3D), suggesting that plasma histidine concentration is affected by gender and/or age in UC patients. The relapse rate of the first quartile within 1 year after remission was ~2.55 times higher than that of the fourth quartile (95% confidence interval: 1.41–4.62) (Table 3).

**Table 2:** P-values and regression coefficient of Cox regression analysis with/without gender- and age-adjustment, and data normalization for each amino acid and CRP.

Amino acids	p value			β
Gender, age adjustment	N	Y	Y	Y
Normalization	N	N	Y	Y
**Thr**	1.97 X 10^−1^	1.85 X 10^−1^	3.45 X 10^−1^	0.1077
**Ser**	3.66 X 10^−1^	4.25 X 10^−1^	4.29 X 10^−1^	0.0871
**Asn**	9.72 X 10^−2^	8.11 X 10^−2^	1.20 X 10^−1^	0.1738
**Glu**	2.54 X 10^−2^	3.37 X 10^−2^	5.11 X 10^−2^	−0.2146
**Gln**	9.36 X 10^−1^	9.53 X 10^−1^	9.14 X 10^−1^	−0.0123
**Pro**	8.34 X 10^−1^	7.40 X 10^−1^	5.87 X 10^−1^	0.0596
**Gly**	7.84 X 10^−1^	6.93 X 10^−1^	8.73 X 10^−1^	−0.0176
**Ala**	5.92 X 10^−1^	5.23 X 10^−1^	7.96 X 10^−1^	0.0294
**Cit**	1.79 X 10^−1^	1.83 X 10^−1^	1.99 X 10^−1^	−0.1433
**a-ABA**	5.97 X 10^−2^	6.69 X 10^−2^	9.81 X 10^−2^	−0.1819
**Val**	3.50 X 10^−1^	4.78 X 10^−1^	3.88 X 10^−1^	−0.0976
**Met**	7.69 X 10^−1^	8.82 X 10^−1^	7.37 X 10^−1^	−0.0375
**Ile**	6.10 X 10^−1^	4.26 X 10^−1^	8.29 X 10^−1^	0.0248
**Leu**	9.21 X 10^−1^	6.93 X 10^−1^	9.26 X 10^−1^	0.0106
**Tyr**	7.93 X 10^−1^	8.79 X 10^−1^	8.04 X 10^−1^	−0.0277
**Phe**	9.84 X 10^−1^	9.08 X 10^−1^	8.81 X 10^−1^	0.0165
**His**	1.12 X 10 ^−2^	1.43 X 10 ^−2^	1.28 X 10 ^−2^	−0.2687
**Trp**	5.14 X 10^−1^	6.03 X 10^−1^	6.83 X 10^−1^	−0.0448
**Orn**	8.27 X 10^−1^	9.42 X 10^−1^	9.07 X 10^−1^	−0.0130
**Lys**	3.87 X 10^−1^	4.81 X 10^−1^	5.07 X 10^−1^	−0.0734
**Arg**	5.98 X 10^−1^	5.51 X 10^−1^	4.37 X 10^−1^	0.0849
**CRP**	5.01 X 10^−1^	5.76 X 10^−1^	4.91 X 10^−1^	0.0753
**TNF-alpha**	6.25 X 10^−2^	6.92 X 10^−2^	8.56 X 10^−2^	0.2102

a-ABA, α-aminobutyric acid.

**Table 3:** Relapse ratio for each quartile by normalized plasma histidine level at each observational term.

Days	1st Quartile	2nd Quartile	3rd Quartile	4th Quartile
**120**	12.5%(11/88)	11.2%(10/89)	2.3%(2/88)	4.5%(4/88)
**180**	14.8%(13/88)	13.5%(12/89)	5.7%(5/88)	9.1%(8/88)
**240**	22.7%(20/88)	16.9%(15/89)	11.4%(10/88)	10.2%(9/88)
**300**	33.0%(29/88)	22.5%(20/89)	14.8%(13/88)	12.5%(11/88)
**365**	35.2%(31/88)	25.8%(23/89)	17.0%(15/88)	14.8%(13/88)

## Discussion

Development of novel biomarkers for prediction of relapse in UC patients in remission has been a long-term goal. It is important for modification of therapy and deciding on monitoring interval, such as timing of endoscopy. Previously, we successfully developed an index using integration of plasma amino acids profile, which could discriminate between IBD patients and healthy controls, as well as between patients with active disease and those in remission. Importantly, plasma histidine concentration was significantly decreased in the patients with active disease compared with those in remission. Plasma histidine is well correlated with serum CRP in CD and with clinical disease activity in both UC and CD [13]. In this study, we tried to develop a novel predictive biomarker by measurement of PFAAs in UC patients in remission. Among PFAAs, we identified that decreased plasma histidine concentration was a risk factor for clinical relapse within 1 year. In comparison between the relapse and non-relapse groups, serum CRP and plasma TNF-alpha levels in patients in remission could not predict relapse within 1 year, while only plasma histidine concentration was significantly decreased in the relapse group. The patients in the lower quartile had a higher risk of relapse. These results suggest that plasma histidine concentration has potential as a predictive marker of relapse in UC patients in remission. Consistent with our studies, lower plasma and urine histidine levels in IBD patients compared with controls have also been reported in several other studies. Plasma amino acids profiling by GC/MS in 13 UC and 21 CD patients showed decreased plasma histidine level in both groups of patients compared with healthy controls [10]. Urinary NMR also showed lower histidine level in urine samples of IBD patients than in healthy controls [9].

As well as histidine, the metabolism of several amino acids is altered in IBD. Gupta et al. reported that the tryptophan catabolism pathway is correlated with CD activity. Decreased serum tryptophan level and increased tryptophan/kynurenine ratio was observed in CD [17]. Indoleamine 2,3-dioxygenase is a tryptophan catabolic enzyme that also regulates immune cell function and intestinal inflammation [18, 19]. Previously, we also observed decreased plasma tryptophan level in active IBD patients [13], however, plasma tryptophan level in UC patients in remission cannot predict clinical relapse within 1 year. Tryptophan may be essential in pathogenesis of CD, which is a typical T helper 1 disease, and is correlated with active inflammation. Citrulline and homocysteine have also been investigated in IBD. Lee et al. reported that plasma citrulline level is inversely correlated with disease activity and CRP in CD [20], while Elkhatib and Buchman reported that plasma citrulline level is not a marker of disease activity in CD [21]. In our study, plasma citrulline level was not a predictive marker of relapse in UC patients in remission. Meta-analysis showed that the risk of hyperhomocysteinemia was significantly higher in IBD patients when compared with controls, although the mechanism of action of hyperhomocysteinemia in IBD, such as correlation with risk of thrombosis, has not been fully investigated [22]. Importantly, except for our study, it has not been demonstrated whether changes in plasma or urine amino acids level are prognostic markers in IBD.

Although the molecular mechanisms of how histidine regulates intestinal inflammation have not been fully investigated, we previously demonstrated that oral administration of histidine ameliorated intestinal inflammation in an interleukin-10-deficient cell transfer mouse model of colitis [23]. *In vitro*, histidine inhibited lipopolysaccharide-induced tumor necrosis factor-alpha production by suppression of nuclear factor (NF)-κB activation in mouse peritoneal macrophages and human monocytes [23]. Hasegawa et al. also reported the inhibitory effects of histidine on the induction of NF-κB activation in THP-1 cells, a human monocytic leukemia cell line, and peripheral blood mononuclear cells [24].

There are limitations to be resolved in the future. Correlation between plasma histidine level and endoscopic findings should be investigated. Comparison with other biomarkers such as fecal calprotectin is another area for research [25, 26].

## Conclusion

In this study, for the first time, we demonstrated that decreased histidine level was associated with increased risk of relapse in UC patients in remission. Metabolomics could be promising for the development of non-invasive predictive biomarkers in IBD.
